# Effect of* Dendrobium officinale* Extraction on Gastric Carcinogenesis in Rats

**DOI:** 10.1155/2016/1213090

**Published:** 2016-12-29

**Authors:** Yi Zhao, Yan Liu, Xi-Ming Lan, Guo-Liang Xu, You-Zhi Sun, Fei Li, Hong-Ning Liu

**Affiliations:** ^1^Research Center for Differentiation and Development of Basic Theory of Traditional Chinese Medicine, Jiangxi University of Traditional Chinese Medicine, Nanchang 330004, China; ^2^Jiangxi Province Key Laboratory of TCM Etiopathogenisis, Nanchang 330004, China; ^3^School of Basic Medical Sciences, Jiangxi University of Traditional Chinese Medicine, Nanchang 330004, China; ^4^State Key Laboratory of Phytochemistry and Plant Resources in West China, Kunming Institute of Botany Chinese Academy of Sciences, Kunming 650201, China

## Abstract

*Dendrobium officinale* (Tie Pi Shi Hu in Chinese) has been widely used to treat different diseases in China. Anticancer effect is one of the important effects of* Dendrobium officinale*. However, the molecular mechanism of its anticancer effect remains unclear. In the present study, gastric carcinogenesis in rats was used to evaluate the effect of* Dendrobium officinale* on cancer, and its pharmacological mechanism was explored.* Dendrobium officinale* extracts (4.8 and 2.4 g/kg) were orally administered to the rats of the gastric carcinogenesis model. Compared with the cancer model group, the high dose of* Dendrobium officinale* extracts significantly inhibited the rate of carcinogenesis. Further analysis revealed that* Dendrobium officinale* extracts could regulate the DNA damage, oxidative stress, and cytokines related with carcinogenesis and induce cell apoptosis in order to prevent gastric cancer.

## 1. Introduction

Gastric cancer is ranked as the fourth most common cause of cancer and as the second most frequent cause of death from cancer in the world [1]. Although the incidence of gastric cancer was decreased in the past ten years, but it was still a main cause of morbidity and mortality [2]. Inhibition, reversion, or extension of each carcinogenic stage will be helpful to prevent from the development of invasive malignancy of gastric cancer.

Chemoprevention was an important approach used to decrease cancer morbidity and mortality. The underlying theory for traditional Chinese medicine (TCM) to treat or prevent cancer is to bring the patient back to a healthy state by modifying multiple cancer-causing events [3]. Many herbal products used for traditional Chinese medicine (TCM) can exert chemopreventive effects [4], such as* Ginkgo biloba* extract [5], tea polyphenol [6], epigallocatechin-3-gallate (EGCG) [7], curcumin [8],* Radix Curcumae* [9], and lycopene [10] which had shown the interventional effect on the progression of gastric precancerous lesions.

*Dendrobium officinale* belongs to the Orchidaceae family, which has been used more than one thousand years in China [11]. The raw and processed* Dendrobium officinale* are used for healthcare products [12]. The major components of* Dendrobium officinale* include water soluble polysaccharides, phenanthrenes, and numerous amino acids [13]. High contents of erianin and chrysotoxene restrained the growth of liver cancer and ascites carcinoma cells [14].* D. officinale* shows the anticancer effects on HeLa S3 human cervical carcinoma cells, HCT-116 cancer cells, and HepG2 liver cancer cells in vitro [15, 16]. In recent years, researchers discovered* D. officinale* also had the preventive effects on the formation of lung metastases and colon carcinogenesis [17, 18]. One previous study revealed that oral administration of* Dendrobium officinale* extraction (DOE) could reduce the incidence in MNNG-initiated rats. The results showed that DOE suppressed abnormal cell proliferation, induced apoptosis, and upregulated proapoptotic gene Bax in gastric cancer. And it could downregulate antiapoptotic gene Bcl-2 and proliferative genes in gastric cancer, such as EGF and EGFR [19]. Nevertheless, the mechanisms of antiproliferative and apoptosis-inducing activity of during MNNG-induced gastric carcinogenesis are not clearly understood. This study will explain the molecular mechanism in which* Dendrobium officinale* mediates the antioxidation, apoptosis, and expressions of DNA oxidative damage, as well as some cytokines related to tumorigenesis.

## 2. Materials and Methods

### 2.1. Materials and Chemicals

*Dendrobium officinale* (lot: SXG131208) was purchased from Zhejiang Shou Xian Gu Pharmaceutical Co., Ltd., and authenticated by Associate Professor Ke zhong Dun (Identification Department of the Jiangxi University of TCM), according to the Pharmacopoeia of the People's Republic of China (2010). The standards schaftoside (lot: 140402), isoschaftoside (lot: 141025), and vicenin 3 (150122) were provided by Weikeqi-Biotech Co. Ltd. (Chengdu, China). N-methyl-N′-nitro-N-nitrosoguanidine (MNNG) (lot: MV340RC) was purchased from Tokyo Chemical Industry. Detection kits for the measurement of glutathione peroxidase (GSH-PX) (lot: 20141124), malondialdehyde (MDA) (lot: 20141107), and superoxide dismutase (SOD) (lot: 20141115) were purchased from Institute of Biological Engineering of NanJing JianCheng (Nanjing, China). And 8-hydroxy-2-deoxyguanosine Elisa kits (8-OHdG) (lot: 20141008) and interleukin 2 (IL-2) (lot: 20141108) were obtained from Neobioscience (Shenzhen, China). Cytokine antibody microarrays (Rat Cytokine Array C2) (lot: 115167103) were supplied by RayBiotech (USA). Antibodies against Bcl-2 (lot: G1315) and Caspase-3 (lot: D2315) were purchased from Santa Cruz Technology (Dallas, TX, USA). Antibodies against Bax (lot: GR151406-13) were purchased from Abcam Technology (Dallas, TX, USA). GAPDH (lot: D16H11) and secondary antibodies were purchased from Cell Signaling Technology. BCA Protein Assay Kit (lot: No. 00121506) was purchased from Cwbiotech Technology. Protein ladder (lot: 00275697) was supplied by Thermo Fisher Scientific.

### 2.2. Animals

Rats used in our trial were supplied by Beijing Wei Tong Li Hua Experimental Animal Research Center. Forty-eight male Wistar rats, 8–10 weeks old, with weights between 110–120 grams were used for this experiment. During pretrial period, rats were kept and fed in groups in the laboratory at room temperature (22°C). The animals were cared for in accordance with* the Guiding Principles for the Care and Use of Animals*.

### 2.3. Preparation and Quality Control of Plant Extracts

The fresh* Dendrobium officinale* was added to 20-fold volume of water and extracted three times by stirring 2 hours one time. The extraction was evaporated using a rotary evaporator and produced the power by spray drying.* Dendrobium officinale* extraction powders (0.1 g) were dissolved with 15% acetonitrile and extracted for 30 min ultrasonic sound. The solution was filtered through 0.45 *μ*m Millipore membrane and the filtrate was collected as sample solution. Standards schaftoside, isoschaftoside, vicenin 3, and naringenin were dissolute in 30% methanol. The final concentrations were 100 *μ*g/mL, 100 *μ*g/mL, 100 *μ*g/mL, and 200 *μ*g/mL, respectively. The chromatographic analysis was performed on C18 column (250 mm × 4.6 mm, 5 *μ*m) by HPLC (Shimadzu LC-20AT). The mobile phase was acetonitrile (A) and 0.1% glacial acetic acid solution (B). The flow rate was 0.8 mL/min. Column temperature was maintained at 30°C. Gradient elution was 0–25 min 14% B, 25–35 min 14%–18% B, and 35–76 min 18%–45% B. Detection wavelength of schaftoside, isoschaftoside, and vicenin 3 was set at 335 nm, and that of naringenin was set at 270 nm. Injection volume was 10 *μ*L for each analysis.

### 2.4. Inhibition Effects of* Dendrobium officinale* on the Progression of N-Methyl-N′-nitro-N-nitrosoguanidine-Induced Gastric Carcinogenesis in Rats

Forty-eight Wistar rats were randomly divided into four groups (*n* = 12): normal control group (Normal), GC model but untreated group (Model), and* Dendrobium officinale* extraction (DOE) group (2.4 g/kg and 4.8 g/kg). Distilled water was given to rats in control group as vehicle. MNNG of 200 mg/kg was orally administered in all rat groups through a catheter after administration of DOE for one hour. MNNG administration was repeated every 10 days for three months (20-21).* D. officinale* was administered daily for three months, as described above. The body weights of rats were recorded weekly. After three months, all animals were sacrificed by intraperitoneal injection of thiopental after overnight fasting. The serum from the inferior vena cava was collected in a tube and centrifuged at 2000*g* for 10 min at 4°C. The stomach was divided into 2 parts. One was used for HE staining, and another was kept at −80°C for Western blot analysis. The protocol for these experiments was approved by the Animal Ethics Committee of Jiangxi University of Traditional Chinese Medicine.

### 2.5. Analysis of 8-OHdG and IL-2 in Serum

The concentrations of 8-OHdG and IL-2 in serum were measured using rat 8-OHdG and IL-2 Elisa kits. Briefly, biotinylated antibody reagent was added to 96-well plates, then supernatants of homogenized serum were added and the plates were incubated at 37°C for 2 h. After washing with phosphate buffered saline (PBS), streptavidin horseradish peroxidase (HRP) solution was added and the plate was incubated for 1 h at room temperature. The absorbance was measured at 450 nm using a microplate reader.

### 2.6. Analysis of SOD, MDA, and GSH-PX in Plasma

SOD activity was measured by xanthine oxidase; the MDA content was measured by the presence of thiobarbituric acid reactive substances (TBARS), and GSH-PX activity was measured by DTNB. All were determined by colorimetric method using corresponding kits.

### 2.7. Microarray Analysis of Cytokines

Cytokine levels in the serum were analyzed with cytokine antibody microarrays following the guideline by manufactory [20]. After development and scan for each sample, a photo of the membranes with optimal image qualities was taken and analyzed by Gel imaging system and image analysis. Finally, results recorded from each membrane were normalized. The signal densities of cytokines were analyzed using a multivariate analysis of variance with post hoc test. *P* values less than 0.05 were considered significant.

### 2.8. Protein Extraction and Western Blot Analysis in the Gastric Tissue

Total stomach tissue protein was obtained with RIPA buffer, and protein concentrations were determined with BCA Protein Assay Kit (Cwbiotech, China). For the Western blot analysis, 50 *μ*g protein was separated by SDS-PAGE and transferred to a nitrocellulose membrane and then blocked with 5% nonfat milk and stained with Bax, Bcl-2, and Caspase-3 primary antibodies (diluted 1 : 1000). The membranes were further incubated with a horseradish peroxidase conjugated secondary antibody (diluted 1 : 10000) for 1 h at room temperature. The blots were washed three times with PBS-Tween and then developed by ECL using a Prolight HRP western blotting detection reagent (Cwbiotech, China).

### 2.9. Statistical Analyses

Data were expressed as mean ± SEM. Statistical differences were determined using one-way repeated measures analysis of variance (ANOVA) followed by Duncan's multiple range as post hoc test. Differences between groups were considered significantly different when *P* value was less than 0.05.

## 3. Result

### 3.1. Quantity Control of DOE by HPLC

**Figure 1:**
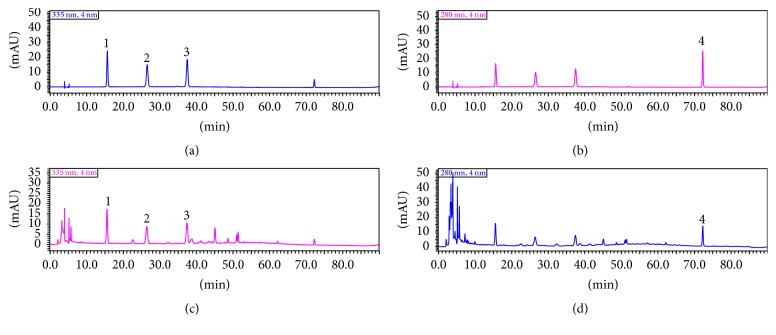
The quality control of DOE by HPLC at 280 *μ*m and 335 *μ*m ((1) vicenin 3, (2) isoschaftoside, (3) schaftoside, and (4) naringenin. (a) standard at 335 *μ*m, (b) standard at 280 *μ*m, (c) sample at 335 *μ*m, and (d) sample at 280 *μ*m.).

As shown in the Figure 1, the concentrations of schaftoside, isoschaftoside, vicenin 3, were in good linear relations between 5–100 *μ*g/mL, and naringenin was 10–200 *μ*g/mL, and the correlation coefficients were 0.9999, 0.9998, 0.9995, and 0.9997, respectively. The results of four components' methodological study demonstrated that the method satisfied the requirements of the determination. According to the method, the concentrations of schaftoside, isoschaftoside, vicenin 3, and naringenin in sample were 6.862 *μ*g/mL, 7.5858 *μ*g/mL, 217.7355 *μ*g/mL, and 12.7902 *μ*g/mL, respectively (Figure 1).

### 3.2. Gastric Cancer Incidence and Body Changes

**Figure 2:**
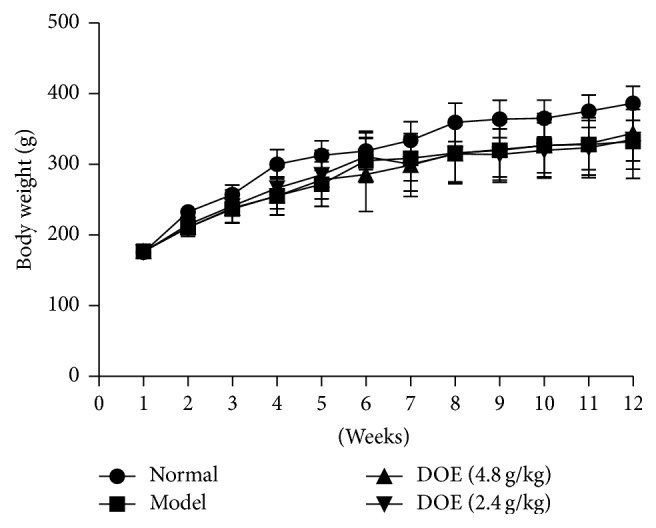
The change of body weight during the experiments (Normal means normal control group; Model means GC model but untreated group; DOE (2.4 g/kg and 4.8 g/kg) means GC model that was given* Dendrobium officinale* extracts (2.4 g/kg and 4.8 g/kg); *n* = 12).

During the whole study period, four rats in positive group and two rats in high-dose group died from aspiration after feeding between 7 and 10 weeks. The results of autopsy showed congestion and bleeding spots in both lungs, while there was hyperkeratosis in forestomach mucosa, but no precancerous lesions or tumor was formed by histological examination. 42 rats survived till week 12 in the end. Histopathological examinations had been reported in the previous study [19], indicating that the gastric cancer incidence in the model group was 41%. DCE could reduce the incidence of cancer and accelerate tumor growth. The body weight showed no significant change between all groups during the periods (Figure 2).

### 3.3. DOE Attenuated Development of Carcinogenesis Associated with 8-OHdG and IL-2 Expression

**Figure 3:**
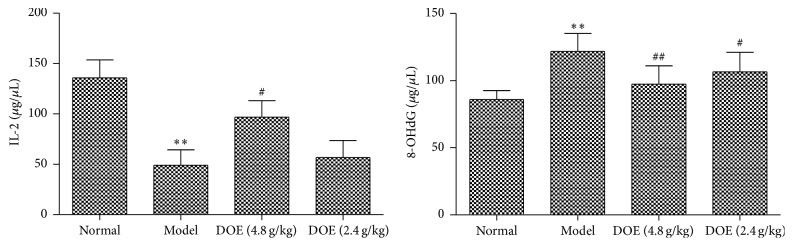
Effect of DOE on 8-OHdG and IL-2 in plasma of gastric cancer rats (Normal means normal control group; Model means GC model but untreated group; DOE (2.4 g/kg and 4.8 g/kg) means GC model that was given* Dendrobium officinale* extracts (2.4 g/kg and 4.8 g/kg). Compared with normal group, ^*∗∗*^*P* < 0.01; compared with model group, ^#^*P* < 0.05 and ^##^*P* < 0.01; *n* = 12).

As shown in Figure 3, the expression of IL-2 and 8-OHdG was significantly different between normal and modern group. It was found that two doses of DOE could suppress 8-OHdG (*P* < 0.01, *P* < 0.05) and high-dose DOE could increase IL-2 significantly compared with the model group (*P* < 0.05).

### 3.4. Analysis of SOD, MDA and GSH-PX in Plasma

**Figure 4:**
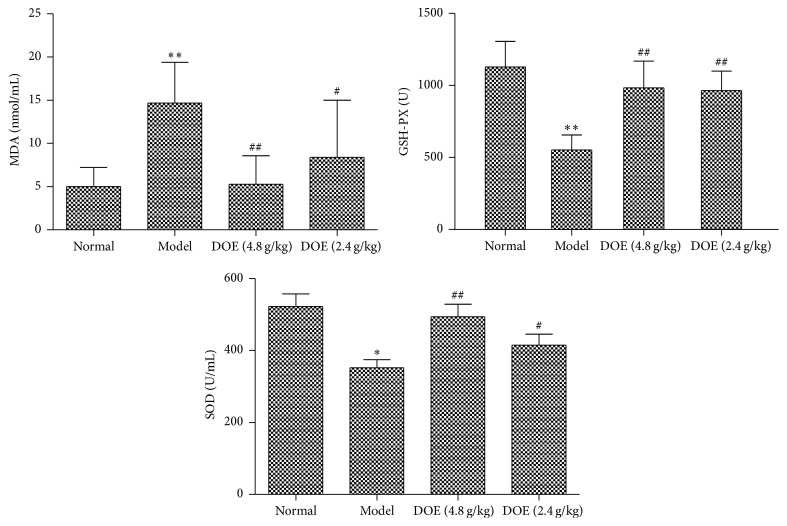
The levels of MDA, SOD, and GSH-PX in serum (Normal means normal control group; Model means GC model but untreated group; DOE (2.4 g/kg and 4.8 g/kg) means GC model that was given* Dendrobium officinale* extracts (2.4 g/kg and 4.8 g/kg). Compared with normal group, ^*∗*^*P* < 0.05 and ^*∗∗*^*P* < 0.01. Compared with model group, ^#^*P* < 0.05 and ^##^*P* < 0.01; *n* = 12).

As shown in Figure 4, compared with the normal group, GSH-PX and SOD activity in the gastric cancer groups was decreased (*P* < 0.01) and the MDA activity was increased markedly (*P* < 0.01). After 12 weeks of treatment, GSH-PX activity was greatly increased in the DOE groups (*P* < 0.01; *P* < 0.01) and also SOD (*P* < 0.01; *P* < 0.05). MDA activity in the DOE groups was decreased significantly (*P* < 0.01; *P* < 0.05).

### 3.5. Relative Expression Levels of Cytokine in Serum Measured from the MNNG-Induced Model Group and DOE Treatment Group

The RayBio Rat Cytokine Array C2 microarrays could allow simultaneous detection of 34 inflammation-related cytokines. Comparing with the normal group, the signal densities of cytokines were upregulated in the MNNG-induced gastric cancer groups, including Activin A, Agrin, IL-1*α* (both *P* < 0.01), ICAM-1 (*P* < 0.001), and TIMP-1 (*P* < 0.05). However, the expression level of IL-10 was reduced (*P* < 0.05). Several other cytokines, including LIX, MCP-1, MMP-8, and CXCL7 were increased, but there was no significant difference. In the DOE treatment group, the signal densities of most inflammatory cytokines were downregulated, including Activin A, Agrin, TIMP-1 (both *P* < 0.01), and ICAM-1 (*P* < 0.001), but IL-10 was increased (*P* < 0.01).

### 3.6. The Protein Expression of Bax, Bcl-2, and Caspase-3 in Rats' Stomach

Because Bax, Bcl-2, and caspase-3 were the key proteins involved in apoptosis, the effect of* D. officinale* on Bax, Bcl-2, and caspase-3 expression was measured. Compared with the model group, the content of Bcl-2 protein quickly recovered after administration of* D. officinale* at 4.8 and 2.4 g/kg for 12 weeks (*P* < 0.01; *P* < 0.05). Additionally, the expression of Bax and Caspase-3 was decreased in the model group. After administration of* D. officinale* at 4.8 and 2.4 g/kg, the contents of Bax protein (*P* < 0.01, *P* < 0.05) and Caspase-3 (*P* < 0.05) were significantly increased.

## 4. Discussion

*Dendrobium officinale* Kimura et Migo is ranked as “the first of the nine Chinese fairy herbs.”* D. officinale* possesses some important medicinal applications, which can maintain tonicity of stomach, promote the body fluid production, reduce peripheral vascular obstruction, and enhance the immune system. It has been commonly used for antitumor activity, antiaging, regulation of blood sugar, treatment of stomach disorders, etc. [21, 22]. Some quality control methods have been reported for* D. officinale*. Zhou et al. [23] using HPLC-DAD-ESI-MS identified eight flavone di-C-glycosides from* D. officinale* leaves in which apigenin and monosaccharide were connected with C-6 and C-8. Chen et al. [24] had quantified bibenzyls, phenanthrenes, and flavanones, which are used for plant identification among the* Dendrobium* species. Chu et al. [25] established a fast and simple method by combining normal and fluorescence microscopy to distinguish* D. officinale*. In our research, we established a method to simultaneously determine the concentrations of schaftoside, isoschaftoside, vicenin 3, and naringenin in* D. officinale* by HPLC-UV. Schaftoside, isoschaftoside, and vicenin 3 showed good linear relations between 5–100 *μ*g/mL, and naringenin was 10–200 *μ*g/mL. The concentrations of schaftoside, isoschaftoside, vicenin 3, and naringenin in sample were 6.862 *μ*g/mL, 7.5858 *μ*g/mL, 217.7355 *μ*g/mL, and 12.7902 *μ*g/mL, respectively (Figure 2).

The generation and increase of ROS and DNA oxidative damage are related to the damage and malignant transformation of gastric mucosa [26]. The antioxidative property of natural agents will show the therapeutic and preventive effect on the process of carcinogenesis, such as the natural antioxidants ginsenosides and curcumin [27, 28]. Our recent work has reported that* D. candidum* could prevent MNNG-induced gastric cancer [19]. Some researchers had reported the preventive effects on other cancers. For example, Wang et al. [18] reported that* D. candidum* Wall ex Lindl. exhibited preventive effects against colon carcinogenesis in mice through increasing Bax, Caspase-3, and Caspase-9 and decreasing Bcl-2.

**Figure 5:**
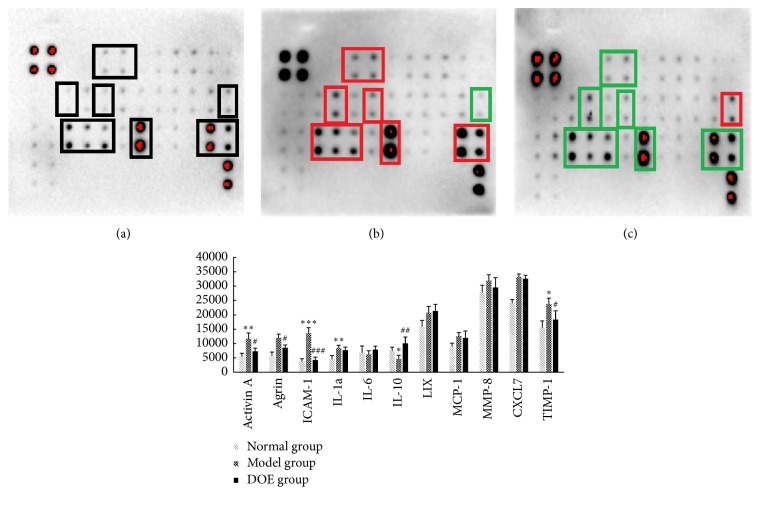
Relative expression levels of cytokine in serum measured from the MNNG-induced model group and DCE treatment group ((a) Normal group, (b) Model group, and (c) DOE (4.8 g/kg). Normal means normal control group; Model means GC model but untreated group; DOE (2.4 g/kg and 4.8 g/kg) means GC model that was given* Dendrobium officinale* extracts (2.4 g/kg and 4.8 g/kg). Compared with normal group, ^*∗*^*P* < 0.05, ^*∗∗*^*P* < 0.01, and ^*∗∗∗*^*P* < 0.001. Compared with model group, ^#^*P* < 0.05, ^##^*P* < 0.01, and ^###^*P* < 0.001. The box in (a), (b), and (c) means the difference between each group; *n* = 4).

Cytokines play an important role in inflammation cell growth and differentiation, which is involved in most disease processes, including cancer. Cytokine array microarrays could detect protein expressions in serum and urine. In our results, the expression of Activin A, Agrin, IL-1*α*, ICAM-1, and TIMP-1 was upregulated, but the expression of IL-10 was reduced. After administration with DOE, the signal densities of most inflammatory cytokines were downregulated, including Activin A, Agrin, TIMP-1, and ICAM-1 (*P* < 0.001) (Figure 5). Also IL-10 was increased. Some studies had reported Activin signal induces growth inhibition and apoptosis through SMAD-dependent pathways [29, 30]. In contrast, several articles have reported that upregulated INHBA expression may promote cancer cell proliferation in several cancers, and the level of Activin A in plasma might be associated with cancer progression in clinical [31–34], In addition, intercellular adhesion molecule 1 (ICAM-1) was upregulated in gastric cancers and cancer progression, which were associated with poor prognostic effect [35, 36]. Tissue inhibitor of matrix metalloproteinase-1 (TIMP-1) is overexpressed in gastric cancer, and the level of TIMP-1 in serum was considered as a marker for prognosis in gastric cancer patients [37]. Therefore, decreasing TIMP activity was a useful method to modulate tumor development and metastasis [38]. In our study, serum ICAM-1 and TIMP-1 levels in MNNG-induced gastric cancer were higher than the normal group*. D. officinale* decreased Activin A, Agrin, TIMP-1 (both *P* < 0.01), and ICAM-1 (*P* < 0.001), and IL-10 was increased (*P* < 0.01). Other cytokines LIX, MCP-1, MMP-8, and CXCL7 were increased in gastric cancer model; however,* D. officinale* did not show the significant effect on these cytokines (Figure 5).

**Figure 6:**
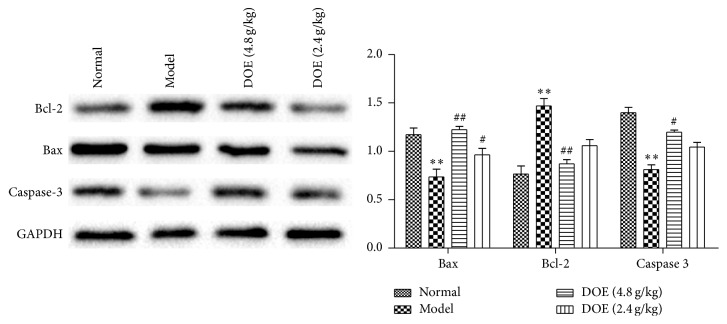
Effects of DOE on the protein expression of Bcl-2, Bax, and Caspase-3 (Normal means normal control group; Model means GC model but untreated group; DOE (2.4 g/kg and 4.8 g/kg) means GC model that was given* Dendrobium officinale* extracts (2.4 g/kg and 4.8 g/kg). Compared with normal group, ^*∗∗*^*P* < 0.01. Compared with model group, ^#^*P* < 0.05 and ^##^*P* < 0.01; *n* = 6).

Induction of apoptosis appears to be associated with their effectiveness in modulating carcinogenic processes [39, 40]. The Bcl 2 family, which includes promoters (Bax and Bid) and inhibitors (Bcl 2 and Bcl xL), is a key regulator in mitochondria mediated apoptosis. In our previous study, we have reported that the gene level of Bax was decreased, whereas Bcl 2 was increased in the model groups. Following treatment with* D. officinale*, Bax was increased and Bcl 2 was decreased. This paper detected the protein expressions of Bax, Bcl 2, and Caspase-3 by western blot, which further confirmed that* D. officinale* at 4.8 and 2.4 g/kg showed strong activity in increasing the expression of Bax (*P* < 0.01, *P* < 0.05) and Caspase-3 (*P* < 0.05). Also it decreased Bcl-2 (*P* < 0.01, *P* < 0.05), therefore promoting apoptosis in preventing the occurrence of gastric cancer (Figure 6).

In conclusion, this study suggests that* D. officinale* could downregulate the expression of MDA and 8-OHdG and upregulate the activity of GSH-PX as well as IL-2 during MNNG-induced gastric tumorigenesis in rats. And* D. officinale* also reduced the level of Activin A, Agrin, IL-1*α*, ICAM-1, and TIMP-1 and upregulated the level of IL-10. Additionally*, D. officinale* increases the protein level of Bax and Caspase-3 and decreases the expression of Bcl-2. The results of the present study coupled with our previous findings indicate that* D. officinale* shows antioxidative effect, modulates some cytokines related to tumorigenesis, and induces apoptosis. These experimental results reveal that* D. officinale* could be developed as a chemopreventive drug for reducing the risk of gastric cancer.

## References

[1] Ferlay J., Shin H.-R., Bray F., Forman D., Mathers C., Parkin D. M. (2010). Estimates of worldwide burden of cancer in 2008: GLOBOCAN 2008. *International Journal of Cancer*.

[2] Ford A. C. (2011). Chemoprevention for gastric cancer. *Best Practice and Research: Clinical Gastroenterology*.

[3] Wang S., Penchala S., Prabhu S., Wang J., Huang Y. (2010). Molecular basis of traditional chinese medicine in cancer chemoprevention. *Current Drug Discovery Technologies*.

[4] Parekh H. S., Liu G., Wei M. Q. (2009). A new dawn for the use of traditional Chinese medicine in cancer therapy. *Molecular Cancer*.

[5] Jiang X. Y., Qian L. P., Zheng X. J., Xia Y. Y., Jiang Y. B., Sun D. Y. (2009). Interventional effect of Ginkgo biloba extract on the progression of gastric precancerous lesions in rats. *Journal of Digestive Diseases*.

[6] Mei Y. Y., Wei D. Zh., Liu J. W. (2005). Modulation effect of tea polyphenol toward *N*-methyl-*N*′-nitro-*N*-nitrosoguanidine-induced precancerous gastric lesion in rats. *The Journal of Nutritional Biochemistry*.

[7] Xu Q., Yang C. H., Liu Q. (2011). Chemopreventive effect of epigallocatechin-3-gallate (EGCG) and folic acid on the N-methyl-N′-nitro-N-nitrosoguanidine (MNNG)-induced gastrointestinal cancer in rat model. *Journal of Digestive Diseases*.

[8] Sintara K., Thong-Ngam D., Patumraj S., Klaikeaw N. (2012). Curcumin attenuates gastric cancer induced by N-methyl-N-nitrosourea and saturated sodium chloride in rats. *Journal of Biomedicine and Biotechnology*.

[9] Lu B., Xu L., Yu L., Zhang L. (2008). Extract of radix curcumae prevents gastric cancer in rats. *Digestion*.

[10] Velmurugan B., Bhuvaneswari V., Nagini S. (2002). Antiperoxidative effects of lycopene during N-methyl-N′-nitro-N-nitrosoguanidine-induced gastric carcinogenesis. *Fitoterapia*.

[11] Weng D. (2003). FAAS determination of trace elements in Dendrobium candidum using suspension sampling with ultrasonic agitation. *Chinese Pharmaceutical Journal*.

[12] Xiao F., Zhang J. Z., Tu Y. L. (2012). First report of Fusarium oxysporum causing wilt of Dendrobium candidum in Zhejiang province, China. *Plant Disease*.

[13] Li J., Li S. X., Huanget D. (2011). Advances in the of resources, constituents and pharmacological effects of Dendrobium officinale. *Science & Technology Review*.

[14] Shao H., Zhang L. Q., Li J. M. (2004). Advances in research of *Dendrobium officinale*. *Chinese Traditional and Herbal Drugs*.

[15] Brew K., Dinakarpandian D., Nagase H. (2000). Tissue inhibitors of metalloproteinases: evolution, structure and function. *Biochimica et Biophysica Acta (BBA)—Protein Structure and Molecular Enzymology*.

[16] Zhao X., Sun P., Qian Y., Suo H. (2014). D. candidum has in vitro anticancer effects in HCT-116 cancer cells and exerts in vivo anti-metastatic effects in mice. *Nutrition Research and Practice*.

[17] Li G., Sun P., Zhou Y., Zhao X., Chen F. (2014). Preventive effects of Dendrobium candidum Wall ex Lindl. On the formation of lung metastases in BALB/c mice injected with 26-M3.1 colon carcinoma cells. *Oncology Letters*.

[18] Wang Q., Sun P., Li G. J., Zhu K., Wang C., Zhao X. (2014). Inhibitory effects of *Dendrobium candidum* Wall ex Lindl. on azoxymethane- and dextran sulfate sodium-induced colon carcinogenesis in C57BL/6 mice. *Oncology Letters*.

[19] Zhao Y., Liu Y., Lan X. M. (2015). Study on inhibition of Dendrobium officinale extract on gastric carcinogenesis. *Chinese Traditional and Herbal Drugs*.

[20] Wu F., Zhang R., Shen X., Lao L. (2014). Preliminary study on pain reduction of monosodium iodoacetate-induced knee osteoarthritis in rats by carbon dioxide laser moxibustion. *Evidence-Based Complementary and Alternative Medicine*.

[21] Zhao M.-M., Zhang G., Zhang D.-W., Hsiao Y.-Y., Guo S.-X. (2013). ESTs analysis reveals putative genes involved in symbiotic seed germination in Dendrobium officinale. *PLoS ONE*.

[22] Wu L. S., Jia M., Chen L. (2016). Cytotoxic and antifungal constituents isolated from the metabolites of endophytic fungus DO14 from *Dendrobium officinale*. *Molecules*.

[23] Zhou G. F., Lu G. Y. (2012). Study on eight flavone c-glycosides in dendrobium officinale leaves and their fragmentation pattern by HPLC-DAD-ESI-MS. *Chinese Pharmaceutical Journal*.

[24] Chen X., Wang F., Wang Y. (2012). Discrimination of the rare medicinal plant Dendrobium officinale based on naringenin, bibenzyl, and polysaccharides. *Science China Life Sciences*.

[25] Chu C., Yin H., Xia L., Cheng D., Yan J., Zhu L. (2014). Discrimination of dendrobium officinale and its common adulterants by combination of normal light and fluorescence microscopy. *Molecules*.

[26] Ni J., Mei M., Sun L. (2012). Oxidative DNA damage and repair in chronic atrophic gastritis and gastric cancer. *Hepato-Gastroenterology*.

[27] Strimpakos A. S., Sharma R. A. (2008). Curcumin: preventive and therapeutic properties in laboratory studies and clinical trials. *Antioxidants & Redox Signaling*.

[28] Choi J.-S., Chun K.-S., Kundu J., Kundu J. K. (2013). Biochemical basis of cancer chemoprevention and/or chemotherapy with ginsenosides. *International Journal of Molecular Medicine*.

[29] Ramachandran A., Marshall E. S., Love D. R., Baguley B. C., Shelling A. N. (2009). Activin is a potent growth suppressor of epithelial ovarian cancer cells. *Cancer Letters*.

[30] Bauer J., Sporn J. C., Cabral J., Gomez J., Jung B. (2012). Effects of Activin and TGF*β* on p21 in Colon cancer. *PLoS ONE*.

[31] Yanagihara K., Takigahira M., Tanaka H. (2008). Establishment and molecular profiling of a novel human pancreatic cancer panel for 5-FU. *Cancer Science*.

[32] Arao T., Fukumoto H., Takeda M., Tamura T., Saijo N., Nishio K. (2004). Small in-frame deletion in the epidermal growth factor receptor as a target for ZD6474. *Cancer Research*.

[33] Mu Y., Gudey S. K., Landström M. (2012). Non-Smad signaling pathways. *Cell & Tissue Research*.

[34] Togashi Y., Kogita A., Sakamoto H. (2005). Activin signal promotes cancer progression and is involved in cachexia in a subset of pancreatic cancer. *Bioorganic & Medicinal Chemistry Letters*.

[35] Fujihara T., Yashiro M., Inoue T. (1999). Decrease in ICAM-1 expression on gastric cancer cells is correlated with lymph node metastasis. *Gastric Cancer*.

[36] Jung W.-C., Jang Y.-J., Kim J.-H. (2012). Expression of intercellular adhesion molecule-1 and E-selectin in gastric cancer and their clinical significance. *Journal of Gastric Cancer*.

[37] Joo Y. E., Seo K. S., Kim H. S., Rew J. S., Park C. S., Kim S. J. (2000). Expression of tissue inhibitors of metalloproteinases (TIMPs) in gastric cancer. *Digestive Diseases & Sciences*.

[38] Zhang S., Li L., Lin J.-Y., Lin H. (2003). Imbalance between expression of matrix metalloproteinase-9 and tissue inhibitor of metalloproteinase-1 in invasiveness and metastasis of human gastric carcinoma. *World Journal of Gastroenterology*.

[39] Sun S.-Y., Hail N., Lotan R. (2004). Apoptosis as a novel target for cancer chemoprevention. *Journal of the National Cancer Institute*.

[40] Kuno T., Tsukamoto T., Hara A., Tanaka T. (2012). Cancer chemoprevention through the induction of apoptosis by natural compounds. *Journal of Biophysical Chemistry*.

